# Chemical Composition, Antioxidant and Antibacterial Activities of *Thymus broussonetii* Boiss and *Thymus capitatus* (L.) Hoffmann and Link Essential Oils

**DOI:** 10.3390/plants11070954

**Published:** 2022-03-31

**Authors:** Imane Tagnaout, Hannou Zerkani, Nadia Hadi, Bouchra El Moumen, Fadoua El Makhoukhi, Mohamed Bouhrim, Rashad Al-Salahi, Fahd A. Nasr, Hamza Mechchate, Touriya Zair

**Affiliations:** 1 Research Team of Chemistry of Bioactive Molecules and the Environment, Laboratory of Innovative Materials and Biotechnology of Natural Resources, Faculty of Sciences, Moulay Ismaïl University, B.P. 11201 Zitoune, Meknes 50070, Morocco; h.zerkani@edu.umi.ac.ma (H.Z.); hadi.nadia@umi.ac.ma (N.H.); elmoumen.bouchra@umi.ac.ma (B.E.M.); elmakhoukhi.fadoua@umi.ac.ma (F.E.M.); mohamed.bouhrim@gmail.com (M.B.); t.zair@umi.ac.ma (T.Z.); 2 Laboratoire Centre Eau, Ressources Naturelles, Environnement Et Développement Durable, Faculty of Sciences, University Mohammed V, Rabat B.P. 8007, Rabat 10000, Morocco; 3 Department of Pharmaceutical Chemistry, College of Pharmacy, King Saud University, Riyadh 11451, Saudi Arabia; ralsalahi@ksu.edu.sa; 4 Department of Pharmacognosy, College of Pharmacy, King Saud University, Riyadh 11451, Saudi Arabia; fnasr@ksu.edu.sa; 5 Laboratory of Inorganic Chemistry, Department of Chemistry, University of Helsinki, P.O. Box 55, FI-00014 Helsinki, Finland

**Keywords:** *Thymus capitatus*, *Thymus broussonetii*, chemical composition, antioxidant, antibacterial activity

## Abstract

*Thymus capitatus* and *Thymus broussonnetii* are two Moroccan endemic medicinal plants used traditionally by the local population. The present study aims to investigate their essential oil chemical composition, antioxidant and antibacterial activities. The chemical composition of the essential oils was determined using the GC-MS analysis, the antioxidant activity assessed using DPPH and FRAP methods while the antimicrobial activity was evaluated against nine bacteria species tested (*Enterococcus faecalis*, *Serratia fonticola*, *Acinetobacter baumannii*, *Klebsiella oxytoca*, sensitive *Klebsiella pneumoniae*, sensitive *Escherichia coli*, resistant *Escherichia coli*, resistant *Staphylococcus aureus* and *Enterobacter aerogenes*). The major identified compounds of *T. capitatus* essential oil where carvacrol (75%) and *p*-cymene (10.58%) while carvacrol (60.79%), thymol (12.9%), *p*-cymene (6.21%) and *γ*-terpinene (4.47%) are the main compounds in *T. broussonnetii* essential oil. The bioactivity of the essential oils of the two species of thyme was explained by their richness in oxygenated monoterpenes known for their great effectiveness with an IC_50_ of 3.48 ± 0.05 and 4.88 ± 0.04 μL/mL and EC_50_ of 0.12 ± 0.01 and 0.20 ± 0.02 μL/mL in the DPPH and FRAP assays, respectively, with an important antibacterial activity. These results encourage the use of these plants as a source of natural antioxidants, and antibacterial additives, to protect food from oxidative damage and to eliminate bacteria that are responsible for nosocomial infections.

## 1. Introduction

Since its first appearance, antibiotic therapy has improved the health aspects of human life by fighting against infectious diseases. Despite the progress of this therapy, we are still living in an era where antibiotic-resistant infections are alarmingly increasing [1]. Antimicrobial resistance is not a new phenomenon; it is considered a way of microbes’ survival by mutation of existing genes, or by acquisition of a resistant gene from another already resistant organism. Thus, the abusive use of antibiotics causes selectiveness, allowing the single genotype most apt to develop [2]. Among the pathogenic bacteria that cause real problems for human health, we find species belonging to the families of Enterobacteriaceae, Pseudomonadaceae and Moraxellaceae, which are almost resistant to all the prescribed antibiotics [3]. Despite the importance of oxidation in the life cycle of cells, oxidative stress is the main cause in the etiology of many human diseases, such as atherosclerosis, arthritis, cardiovascular disorders, Alzheimer and cancer [4]. Meanwhile, synthetic antioxidants such as butylatedhydroxytoluene (BHT) used in the food industry for food stabilization have carcinogenic effects [5]. To solve these problems, the use of medicinal plants with antioxidant and antimicrobial properties is one of the most interesting approaches to adopt. Recently, essential oils (EOs) have received increasing attention due to their therapeutic properties, which are beneficial to health, and are generally considered as safe according to the United States Agency for Food and Drug Products [6]. The EOs are natural substances that are composed of volatile secondary metabolites. They are characterized by a strong odor, and they consist of a complex mixture of compounds including: monoterpene and sesquiterpenes hydrocarbons, their oxygenated derivatives (e.g., alcohols, aldehydes, ketones and ethers), several derivatives of phenyl propane, phenols and various volatile organic compounds [7,8]. The EOs chemical profile is always characterized by the presence of one, or a few majority compounds at high concentrations compared to others, which are minority compounds. Several studies have linked the EOs antimicrobial and antioxidant activities to both the functional groups of the majority compounds and the minority compounds possible synergistic or antagonistic effects [9,10]. The EOs are recognized to have several biological activities, such as antibacterial, antifungal and insecticide [11]. Various studies reported the antimicrobial, and antioxidant activities of the EOs of certain medicinal plants [6,12,13,14]. Consequently, the EOs antimicrobial and antioxidant activities can differ according to variations in the chemical composition [15,16]. Thus, the study of the EOs chemical composition, and the evaluation of their biological activities are necessary to confirm their use as preservatives in food, pharmaceutical and cosmetic fields. The genus *Thymus* is considered to be one of the eight most important genera in the Lamiaceae family comprising around 215 species, whichare native to the Mediterranean basin [17]. Thyme’s medicinal and aromatic properties have made it one of the most popular genera in the world. Besides, its essential oils are widely used to flavor and preserve several food products. The majority of these oils are characterized by their richness in oxygenated monoterpenes, in particular phenolic compounds such as thymol and its isomer carvacrol, accompanied by other more or less biologically active compounds such as: eugenol, *p*-cymene, terpinene, linalool, geraniol and borneol [18]. Several studies linked *Thymus* EO chemical composition to its antimicrobial and antioxidant activities [19]. *T. capitatus* (Synonym *Thymbra capitata* (L.) Cav. or Corido *thymus capitatus*) is an endemic Mediterranean plant [20]. In Morocco, this species grows in the thermo-Mediterranean and meso-Mediterranean vegetation stages. It is a much-branched subshrub of 20–50 cm height, covered with whorled leaves, sessile, deciduous if dry, glabrous but slightly ciliated at the base; the light pink flowers, spotted with mauve, grouped in dense ovoid flower heads, differ from other Thymes by the shape of the dorsally flattened calyx [21]. It is used as a food preservative for meat and fish [22]. *T. broussonetii*, endemic to Morocco, is an erect shrub with erect stems. It leaves are stem-like, broadly ovate lanceolate and punctuated on both sides. It flowers are wider and often purple colored, it is characterized by dense male inflorescences, its calyx is bilabiated, the upper lip not very toothed, and its pink corolla, having 2–3 times the length of the calyx, contains a narrow but clearly protruding tube [21]. The severe exploitation to which this species is exposed, can lead to its rarefaction and/or disappearance [23]. There is no study on the variation in the chemical composition of *T. broussonetii* EO. The Mediterranean flora in general, and Morocco in particular, gathers a diversified flora with an endemism rate equal to 878 species, including several species of plants, that are little or not studied at all [24,25]. The present study is a contribution to the chemical and biological valorization of the EOs of two endemic plant species growing in Morocco: *Thymus capitatus* and *Thymus broussonnetii* Boiss.

## 2. Results and Discussion

### 2.1. Essential Oil Yields

In industry, hydrodistillation was considered as the most practical extraction method. Average yields of essential oils were calculated based on the dry plant matter of the aerial part of each plant. Indeed, the leaves and flowering tops of *T. capitatus* from the Khemisset region in the center of Morocco provide an EO yield of 2.6 ± 0.1%. It is relatively high compared to that of northern Morocco (Tetouan) whose yield was 2.05% [26], as well as that of Tunisia with 1.26% [27] and 2.52 mL/g [28]. According to Bounatirou et al. the essential oil yield of *T. capitatus* reaches its maximum during the post-flowering phase [29]. *T. broussonetii* EO yield is 3.35 ± 0.05%; it appears richer in EO than *T. capitatus*. This result is much higher than those reported by several researchers [12,30,31]. From the results reported in various studies and those obtained in our work, we can conclude that: firstly, the EO yields of the studied *T. capitatus* and *T. broussonetii* are very high, which will allow their exploitation on an industrial scale. Secondly, several factors influence the EO yield, such as the harvest period and its place, the drying and extraction techniques as well as the age of the plant [32].

### 2.2. Chemical Composition of T. capitatus and T. broussonetii EOs

**Figure 1:**
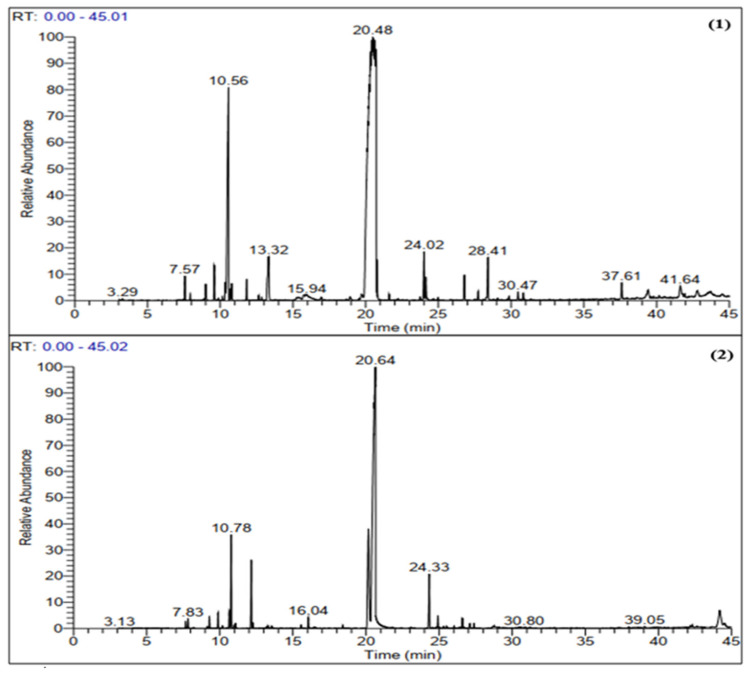
GC-MS chromatogram of *T. capitatus* (**1**) and *T.broussonetii* (**2**) EOs.

**Figure 2:**
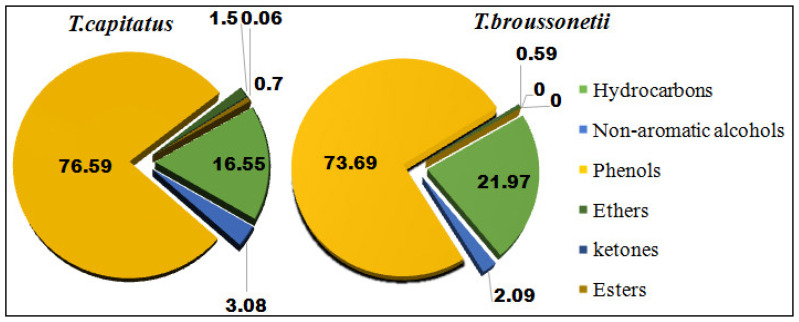
Distribution of the main chemical families of *T. capitatus* and *T. broussonetii* EOs.

The chemical compositions of *T. capitatus* and *T. broussonetii* EOs were determined by gas chromatography coupled with mass spectrometry (GC-SM). The analysis of the chromatograms (Figure 1) showed that the chemical profile of our EOs varies from those of other origins, by the existence of qualitative and quantitative differences of the individual components. The identification of the chemical composition of the EOs (Table 1) revealed a determined number of compounds representing 98.48% of the total composition for *T. capitatus* and 98.34% of the total composition for *T. broussonetii. T. capitatus* EO is rich in carvacrol (75%), *p*-cymene (10.58%), linalool (2.91%), (*E*)-caryophyllene (1.61%) and epoxy caryophyllene (1.5%). As for *T. broussonetii* EO, it is characterized by a high percentage of carvacrol (60.79%), thymol (12.9%), *p*-cymene (6.21%), *γ*-terpinene (4.47%) and (*E*)-caryophyllene (4.15%). The EOs of these two species (*T. capitatus* and *T. broussonetii*), which belong to the genus Thymus, are strongly dominated by oxygenated monoterpenes (79.3% and 75.98%, respectively) and hydrocarbon monoterpenes (13.87% and 14.84% respectively). In comparison, sesquiterpenes compounds are present in *broussonetii* thyme oil (7.52%) more than in capitized thyme (5.31%). It should be noted that carvacrol was the main constituent of the two studied thyme oils. In addition, it was revealed that phenols were particularly abundant in *T. capitatus* essential oil (76.59%), which was richer in carvacrol (75%) than *T. broussonetii* EO (60.79%). The *p*-cymene content was higher in *T. capitatus* essential oil (10.58% against 6.21%), and the contents of thymol, (*E*)-caryophyllene and *γ*-terpinene in *T. capitatus* EO represent only 0.18%, 0.53% and 1.61%, respectively, while they represent 12.9%, 4.15% and 4.47% in *T. broussonetii* EO. *T. capitatus* EO from northern Morocco (Tetouan), is composed mainly of carvacrol and other compounds with relatively low contents such as, *p*-cymene, *γ*-terpinene, linalool, *β*-caryophyllene and *β*-pinene [26]. El Ouariachi et al., (2011) confirmed the same composition through their studies for the same plant from the same origin [33]. In Tunisia, several authors were reported, that the essential oil of *T. capitatus* was mainly composed of carvacrol (61.6–83%), *p*-cymene (5–17%), *γ*-terpinene (2-14%) and *β*-caryophyllene (1–4%) but this composition changes depending on the location and the growing season [29]. Similarly, the EOs of *T. capitatus* from Sicily [34,35], Albania [36], Greece and Portugal [37] are characterized by a high content of carvacrol. While, the *T. capitatus* EO of Sardinia (Italy) was dominated by thymol (29.3%) and *p*-cymene (26.4%); while, carvacrol represents only (10.8%) [38]. In another study, it was revealed that, *T. capitatus* essential oil collected from the south of Tunisia was richer in thymol with a rate of 89% [39]. Finally, the Turkish *T. capitatus* essential oil was characterized by a large amount of carvacrol (35.6%), *p*-cymene (26.4%) and thymol (18.6 %) [40]. In this work, compared to several studies that were previously conducted in Morocco, the chemical composition of the studied *T. broussonetii* EO shows qualitative and quantitative differences, El Ouariachi et al. showed that thyme harvested from northern Morocco (Al Hoceïma region) has borneol (27.6%) as major component, followed by *p*-cymene (20.9%), and carvacrol (15.7%) [41]. While Belaqziz and his collaborators, found that *p*-cymene (21%) is the major compound in thyme harvested from southwest Morocco (around Essaouira), followed by borneol (16.5%), α-pinene (11.8%) and thymol (11.3%) [42]. In the same region, Saad et al. identified thymol (39.64%) as the most abundant compound accompanied by carvacrol (21.31%) and borneol (20.13%) in *T. broussonetii* EO [43]. Another study conducted by Alaoui Jamali et al. revealed that the main constituents of the essential oil of *T. broussonetii* are carvacrol (43.4%), thymol (12.3%), γ-terpinene (8.9%), borneol (8.5%) and *p*-cymene (5.2%) [12]. El Bouzidi et al., in the EO of wild broussonetii thyme identified the same composition [14]. While the oil obtained from cultivated broussonetii thyme is characterized by a higher level of carvacrol (60.8%), *p*-cymene (7.2%) and α-pinene (6.5%). In addition, Tantaoui et al. showed that the major element in the essential oil of *T. broussonetii* was carvacrol (47.8%), accompanied by considerable quantities of hydrocarbons [44]. Based on the results set out in Figure 2, we find that the essential oils of these two species (*T. capitatus* and *T. broussonetii*) are rich in phenols (76.59% and 73.69%) and hydrocarbons (16.55% and 21.97%). We also note the presence, in small proportion, of non-aromatic alcohols (3.08%, 2.09%), ethers (1.5%, 0.59%). As for esters (0.7%) and ketones (0.06%), they are only present in *T. capitatus* EO. However, the level of compounds identified in the analyzed EOs confirms previous literature findings. The difference in chemical composition is caused by several factors, such as species, origin, growth stage, environmental influences and genetic background. Generally, the influence of these factors on the biosynthetic pathways induces changes in qualitative and quantitative terms of the characteristic majority compounds, which lead to the existence of different chemotypes distinguishing EOs from different origins [19,45].

### 2.3. Antioxidant Activity of T. capitatus and T. broussonetii Essential Oils

**Figure 3:**
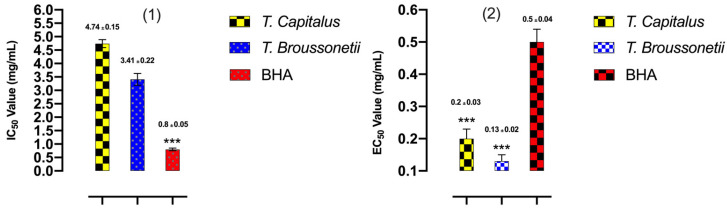
IC_50_ and EC_50_ of *T. capitatus and T. broussonetii* EOs and of BHA standard measured by DPPH (**1**) and FRAP (**2**) methods. Data are expressed as mean ± SD. The experiment was performed in a minimum of three replicates. *** *p* < 0.001 compared to the samples (**1**); *** *p* < 0.001 compared to the control BHA (**2**).

**Figure 4:**
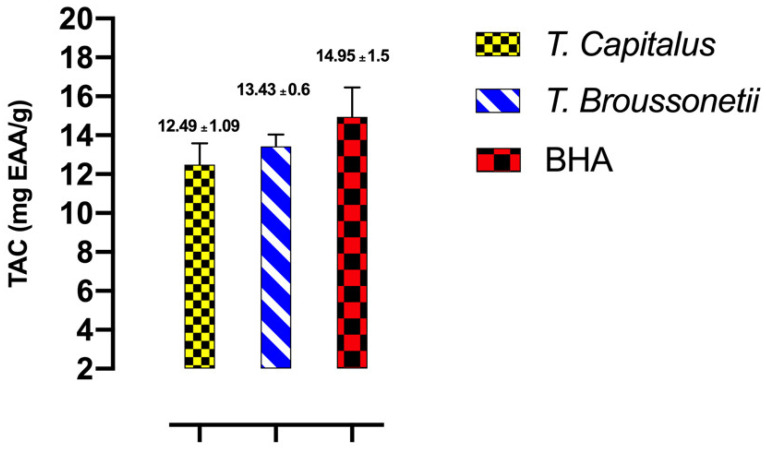
Total antioxidant capacity (Abs. at 695 nm) of *T. capitatus* and *T. broussonetii* essential oils; with the regression curve of ascorbic acid. Data are expressed as mean ± SD. The experiment was performed in a minimum of three replicates.

The antioxidant activities of *T. capitatus* and *T. broussonetii* EOs and of standard (BHA) were evaluated using three methods, DPPH, FRAP and TCA. The results were illustrated in Figure 3 and Figure 4. The EOs are qualified as natural antioxidants, because of their ability to reduce and/or prevent the formation of free radicals. In the food industry, EOs are considered potential substitutes to synthetic antioxidants for food preservation [46]. In this work, the antioxidant activity of the studied EOs is determined by two different methods, DPPH and FRAP. In the presence of antioxidants, the purple DPPH radical transforms into a stable yellow molecule. The degree of discoloration may indicate the ability to trap free radicals. According to the DPPH method, it turns out that the studied EOs have the capacity to reduce the free radicals of DPPH. At a concentration of 0.12 μL/mL, the BHA standard presented a percentage of DPPH free radical inhibition (89.5%) that is higher than the studied EOs (*T. broussonetii* (78.4%), *T. capitatus* (75%)). Indeed, the studied essential EOs showed a dose-dependent anti-free radical activity, the more we increase the concentration, the more the percentage of inhibition increases until reaching a plateau. This phenomenon is explained by the transfer of single electrons located in the external orbital of DPPH. At a certain concentration, the antioxidant reacts with the radical, when we increase the concentration; the antioxidant activity remains constant since this is accompanied by the saturation of the electronic layers of the radical. According to the IC_50_ values presented in Figure 3, the antioxidant power of BHA (IC_50_ = 0.82 ± 0.01 mg/mL) is greater than that of the studied thyme. Among the tested EOs, the most active is *T. broussonetii* essential oil with an IC_50_ of around 3.48 ± 0.05 μL/mL. The high activity of *T. broussonetii* essential oil has already been indicated in several works including El Ouariachi et al., Alaoui Jamali et al. and El Bouzidi et al., [14,31,41,47]. *T. capitatus* EO also demonstrated a high antioxidant power with an IC_50_ of 4.88 ± 0.04 μL/mL. This observation was reported by, Amarti et al., Bounatirou et al. and Zaïri et al. [29,48,49], the latter showed that this essential oil harvested during the flowering phase and after flowering, has more antioxidant power compared to that of the vegetative period before flowering. For the FRAP method, the presence of reducing agents in the medium results in the reduction of the complex Fe^3+^(ferric cyanide) of yellow color, to the ferrous form of greenish blue color, by the donation of an electron. The increase in absorbance at 700 nm indicates an increase in the iron reduction capacity. According to the results of the FRAP tests illustrated in Figure 3, it appears that the EO of *T. broussonetii* has a higher antioxidant power (EC_50_ = 0.13 ± 0.01 mg/mL) than that of standard BHA (EC_50_ = 0.5 ± 0.01 mg/mL). It is a very promising result for food preservation. since the undesirable side effects of synthetic antioxidants are widely known, namely liver damage or carcinogenesis [50]. While, the essential oil of *T. capitatus* that has an EC_50_ of about 0.20 ± 0.02 μL/mL is considered close to BHA standard. The results obtained by the FRAP method confirm the antioxidant potential of the studied EOs. In addition, tests show that the oils have a good affinity with Fe^3+^ ions. The phosphomolybdenum method is quantitative since the total antioxidant activity is expressed as the number of equivalents of ascorbic acid. EOs of tested species of genera *Thymus* revealed good total antioxidant capacity. Results showed that *T. broussonetii* EO exhibited highest TAC with 12.54 mg AAE/g of EO) followed by *T. capitatus* EO (11.74 mg AAE/g of EO). As compared to the synthetic antioxidant BHA (14.33 mg AAE/g), all the oils exhibited approximate total antioxidant capacity (Figure 4). Furthermore, the results suggest that essential oils from Thymus species could be used as natural antioxidants in food systems. Generally, the antioxidant activity is the result of the interaction between all the chemical components of the EO (alcohols, phenols, and terpene and ketone compounds), acting in an antagonistic or synergistic way. In fact, several studies have highlighted the correlation between the phenol content and the antioxidant capacity of plants [12,16]. According to this study, it is evident that the great antioxidant capacity of the studied EOs of *Thymus* (*capitatus* and *broussonetii*) is linked respectively to their richness in oxygenated monoterpenes (79.3% and 75.98%) and in monoterpene hydrocarbons (13.87% and 14.84%), which have a large percentage of phenols (76.59%, 73.79%) and hydrocarbons (16.55, 21.97%). In addition, the presence of minority compounds such as aromatic alcohols, ethers, ketones and esters in the studied EOs, can influence the antioxidant activity. According to the literature, essential oils composed of monoterpenes and/or oxygenated sesquiterpenes are known for their great antioxidant properties [51]. These compounds are capable of trapping free radicals by their phenolic hydroxyl groups. In the case of the studied thyme species, their antioxidant potential is linked to the high content of phenolic compounds such as carvacrol and thymol [16,52].

### 2.4. Antibacterial Activities of T. capitatus and T. broussonetii Essential Oils

#### 2.4.1. Diffusion Method

The antibacterial activity of the EOs can be classified into three levels: (i) weak activity (zone of inhibition ≤ 12 mm), (ii) moderate activity (12 mm < zone of inhibition < 20 mm) and (iii) strong activity (inhibition zone ≥ 20 mm). The results of the antibacterial activity of *T. capitatus* and *T. broussonetii* EOs were presented in Table 2. Diffusion tests carried out with a volume of 5 μL, showed that the tested EOs have an antibacterial activity, against most of the examined bacteria with diameters of inhibition zones ranging from 8.55 ± 0.21 to 49.9 ± 0.14 mm. The EOs isolated from the aerial parts of the studied thyme, showed a strong antibacterial activity against all the tested strains, in particular against *K. oxytoca*, *S. fonticola*, sensitive *E. coli*, *E. aerogenes*, *E. faecalis* and *A. baumannii*, having inhibition zones that vary from 20.70 ± 1.20 mm to 49.9 ± 0.14 mm. While moderate activity was observed against sensitive *K. pneumoniae* and resistant *E. coli*, with inhibition zones ranging from 14.15 ± 0.15 mm to 19.95 ± 0.07 mm. *S. aureus* was more resistant to the tested EOs. Compared with the antibiotics timentin TIM85, cefoxitin FOX30 and piperacillin PRL100 used as controls, the EOs of the studied thymes showed a stronger inhibitory action. The strains that showed resistance to the action of antibiotics are vulnerable to the action of the two types of essences of *T. capitatus* and *T. broussonetii*; this is the case of *E. faecalis*, *S. fonticola*, *K. oxytoca*, and susceptible *E. coli* and *E. aerogenes*. Similar results were reported previously for the EOs of T. capitatus from different origins [53,54,55,56]. According to Bounatirou et al., *T. capitatus* EOs harvested in the flowering and post-flowering phase have better antibacterial activity against *S. aureus* with an inhibition zone of 17.4 mm. While the thyme essential oil harvested in the vegetative phase before flowering, shows no antibacterial activity against the tested microorganisms [29]. Thus, the essential oil of Tunisian *T. capitatus* revealed an important antibacterial potential against Gram-negative bacteria in particular *E. coli* and *K. pneumoniae* giving inhibition zones of 30 mm and 20 mm, respectively [39]. Regarding *T. broussonetii*, its EO also showed an interesting antibacterial power with a dose of 10 μL against *E. coli* and *S. aureus* with inhibition zones of 21 mm and 19 mm [42]. El Bouzidi et al., noted that the EO of broussonetii thyme with the same dose gave inhibition zones of 30.17 mm and 35 mm against *E. coli* and *S. aureus*, respectively [14]. Also, Fadli and his collaborators’ study, showed an antibacterial effect of the EO of *T. broussonetii* with inhibition zones of 22.6 mm, 25.3 mm and 22.7 mm against the *S. aureus*, *E. coli* and *K. pneumoniae* strains respectively [30].

#### 2.4.2. Determination of Minimum Inhibitory (MIC) and Bactericidal (MBC) Concentrations in μL/mL for Bacterial Strains

The antimicrobial activities of the studied EOs were evaluated according to the diameter of inhibition, which indicates the power of inhibition of the EO against the tested strains. The results of the tests were presented in Table 3. According to the obtained results, the MIC values confirm the results of the diffusion method. In these tests, the essential oil of *T. capitatus* was more active than that of *T. broussonetii*. The lowest MIC manifested by the tested EOs was 2 μL/mL against sensitive *E. coli* and sensitive *K. pneumoniae*. The most resistant strain was *S. aureus*, inhibited at a MIC of 16 μL/mL by *T. capitatus* EO and 32 μL/mL by *T. broussonetii* EO, while *E. coli* resistant was inhibited at a MIC of 8 μL/mL for *T. capitatus* and 16 μL/mL for *T. broussonetii*. The results of *T. capitatus* and *T. broussonetii* were the same against the *E. faecalis*, *S. fonticola*, *K. oxytoca* and *A. baumannii* strains (MIC = 4 μL/mL). While, the *E. aerogenes* strain was inhibited at 4 μL/mL by *T. capitatus* EO and at 8 μL/mL by *T. broussonetii* EO. Based on the MBC/MIC report, the EOs of the studied Thymes reported a bactericidal effect towards the bacteria of *E. aerogenes*, *K. pneumoniae* sensitive, *E. faecalis*, *S. fonticola*, *K. oxytoca*, *A. baumannii*, *Staphylococcus aureus*, Resistant and wild *Escherichia coli*. These results show the bactericidal power of *T. capitatus* and *T. broussonetii* EOs against the studied bacteria. Based on these results, the essential oils of *T. capitatus* and *T. broussonetii* could potentially be used as natural preservatives in food against the well-known causative agents of foodborne illness such as *S. aureus* and *E. coli* [57]. In the literature, the essential oils from the leaves of the thymus genus are known for their antibacterial power, which is linked to the origin of the plants and to the essential oil composition [14,30,48,49]. In this work, the EOs inhibitory activities of the studied Thymes are probably due mainly to the phenolic action of carvacrol and thymol (two oxygenated monoterpenes) [58,59]. Several authors explained the antimicrobial activity of carvacrol and thymol against *E. coli* in vitro experiments [60,61]. Carvacrol is considered a biocide, causing disruption of the bacterial membrane, which leads to leaks of intracellular ATP and potassium ions and finally, death of the cell [62]. However, the dominance of oxygenated monoterpene compounds does not necessarily mean, the presence of better antibacterial effects for most of the analyzed strains, because possible synergistic and/or antagonistic effects of the minority compounds present in the oil should also be taken into consideration.

## 3. Material and Methods

### 3.1. Materials and Reagents

Timentin (TIM85), Cefoxitin (FOX30) and Piperacillin (PRL100), were purchased from Sigma Aldrich (St-Quentin Fallavier, France). All chemicals and solvents were of highly analytical grade and were used as received from the supplier without further purification.

### 3.2. Plant Material

**Figure 5:**
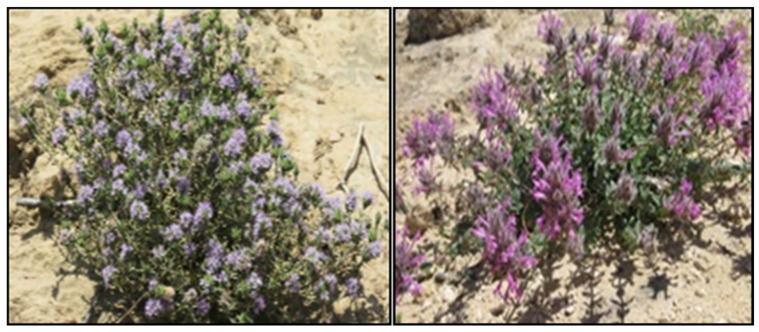
Morphological profile of *T. capitatus* (**Left**) and *T. broussonetii* (**Right**).

Samples of *Thymus capitatus*, and *Thymus broussonetii* Boiss aerial parts (stems, leaves and flowers) were collected in May 2018, in the Khemisset region in the center of Morocco. The species identification was carried out at the plant ecology laboratory in the Scientific Institute of Rabat. The different parts of the plants were dried in the shade for 13 days (Figure 5).

### 3.3. Bacterial Strains

The antibacterial activity in this study was tested on nine bacterial strains that are frequent in human pathology. They belong to the Gram-negative category. The bacterial strains used were the sensitive *Escherichia coli*, resistant *Escherichia coli*, *Enterobacter aerogenes*, sensitive *Klebsiella pneumonia*, *Enterococcus faecalis*, *Serratia fonticola*, *Acinetobacter baumannii*, *Klebsiella oxytoca* and *Staphylococcus aureus*. They were isolated from the patients’ biological fluids, and then preserved by subculturing on specific agar medium in the laboratory of bacteriology in Mohammed V hospital in Meknes.

### 3.4. Extraction of Essential Oils

The essential oils were extracted by hydrodistillation using a Clevenger type apparatus (VWR, Radnor, PA, USA) for three hours. The process was repeated three times for each 100 g of the plant sample. The obtained essential oils were dried over anhydrous sodium sulfate. They were then stored at a temperature of 4 °C in the dark until they were used.

### 3.5. Chromatographic Analysis of Essential Oils

The chromatographic analysis of *T. capitatus* and *T. broussonetii* essential oils was carried out using a gas chromatograph of the Thermo Electron type (Trace GC Ultra) (conquerscientific, Poway, CA, USA) coupled to a mass spectrometer system of the Thermo Electron Trace MS type. (Thermo Electron: Trace Ultra GC, Polaris Q MS) (conquerscientific, Poway, CA, USA), the fragmentation was achieved by an electronic impact intensity of 70 eV. The chromatograph was equipped with a DB-5 column (5% phenyl-methyl-siloxane) (30 m × 0.25 mm × 0.25 μm film thickness), a flame ionization detector (FID) supplied by a mixture of He gas /Air. The column temperature was programmed at a speed of 4 °C/min from 50 to 200 °C for 5 min. The injection mode is split (leak rate: 1/70, flow rate mL/min), the carrier gas used was nitrogen with a flow rate of 1 mL/min. The identification of the essential oil compounds was carried out by comparison of their Kováts index (KI) and Adams with those of the reference products known in the literature [63,64]. The mass spectra and indices of each of these compounds were also compared with those of the databases cited above [65,66]. The Kováts index compares the retention time of any product with that of a linear alkane of the same carbon number.

### 3.6. Essential Oils Antioxidant Activity

#### 3.6.1. DPPH Anti-Free Radical Method

The *T. capitatus* and *T. broussonetii* EOs antioxidant activity was established by the method using DPPH (1,1-diphenyl-2-picrylhydrazyl) as a relatively stable radical [67]. The DPPH solution was prepared by dissolving 2.4 mg of DPPH powder in 100 mL of ethanol. Different concentrations of *T. capitatus* and *T. broussonetii* EOs were prepared by dilution in absolute ethanol (1–20 μL/mL). The test was carried out by mixing 200 μL of each concentration with 2.8 mL of DPPH solution. These same concentrations were prepared with butylhydroxyanisole (BHA) to serve as positive controls. In addition, a blank was made with absolute ethanol alone. The samples were then left in the dark for 30 min. The absorbance of the mixtures was measured at 517 nm. The results were expressed as an inhibition percentage of DPPH free radical (IP %):

IP (%) = (A_control_ − A_Sample_)/A_control_ × 100

IP%: inhibition percentage of DPPH free radical.

A_control_: Absorbance of the solution without the samples.

A_Sample_: Absorbance of the solution in presence of the samples (essential oils or BHA).

The graph representing the inhibition percentage as a function of the samples concentrations made it possible to determine IC_50_, which is the required concentration to inhibit or reduce 50% of the initial concentration of DPPH. It was determined graphically by linear regression. Since there is no absolute measure of the antioxidant capacity of a compound, the results were often reported in relation to a reference antioxidant, such as BHA.

#### 3.6.2. Ferric Reducing Antioxidant Power Method

The power of phenolic extracts to reduce ferric iron (Fe^3+^) present in the potassium ferricyanide complex into ferrous iron (Fe^2+^) was determined according to the method described by Zovko Koncic et al. [68]. In test tubes, 1 mL of each essential oil of plant was diluted in absolute ethanol at different concentrations (1–20 μL/mL). Then, each tube was mixed with 2.5 mL of a phosphate buffer solution (0.2 M, pH = 6.6) and 2.5 mL of a potassium ferricyanide solution K_3_Fe(CN)_6_ at 1%. The obtained solution was incubated in a water bath at 50 °C for 20 min. Then 2.5 mL of 10% trichloroacetic acid was added to stop the reaction processes. The whole solution was centrifuged at 3000 revolutions per minute for 10 min. After, 2.5 mL of the supernatant of each concentration was mixed with 2.5 mL of distilled water, and 0.5 mL of the aqueous FeCl_3_ solution (0.1%). The absorbance of the reaction medium was measured at 700 nm against a similarly prepared blank, replacing the essential oil with absolute ethanol in order to calibrate the apparatus (UV-VIS spectrophotometer (VWR, Radnor, PA, USA). The positive control was represented by a solution of standard antioxidants; the absorbance of BHA was measured under the same conditions as the samples. An increase in absorbance corresponds to an increase in the reducing power of the tested EOs.

#### 3.6.3. Total Antioxidant Capacity

The total antioxidant capacity (TAC) of the essential oils was evaluated according to the method described by Prieto et al. [68]. An aliquot of 100 μL of each essential oil diluted in ethanol was combined with 1 mL of reagent solution (0.6 M sulfuric acid, 28 mM sodium phosphate, and 4 mM ammonium molybdate). The tubes were incubated in a boiling water bath at 95 °C for 90 min. After the samples were cooled at room temperature, the absorbance of the aqueous solution of each sample was measured at 695 nm in the spectrophotometer (Thermo scientific, UV1-V7.09 (VWR, Radnor, PA, USA)). The results were expressed as equivalents of ascorbic acid (mg/g) of oil. Moreover, the BHA was used as a control positive.

### 3.7. Essential Oils Antibacterial Activity

The antibacterial activity was determined using the agar disk diffusion method (Mueller Hinton Agar). The microorganisms resulting from the growth on the nutritive agar incubated at 37 °C for 18 h were suspended in a saline solution (NaCl) at 0.9%, and adjusted to a turbidity of 0.5 Mac Farland standard (108CFU/mL). The suspension was inoculated in 90 mm diameter Petri dishes using a sterile, non-toxic wooden cotton swab. The EOs were dissolved in 0.5% (*v*/*v*) of dimethyl sulfoxide (DMSO), which does not affect the growth of bacteria according to our experiences. Sterile paper disks of 6 mm in diameter impregnated with 5 μL of the studied EOs were placed in the petri dishes and incubated for 24 h at 37 °C. Commercial antibiotics timentin TIM85, cefoxitin FOX30 and piperacillin PRL100 were used as a positive reference to determine the sensitivity of the tested strains. Finally, the diameters of the inhibition zones (DIZ) were measured in order to determine the studied EOs antibacterial activities.

### 3.8. Determination of the Minimum Inhibitory Concentration (MIC) and the Minimum Bactericidal Concentration (MBC) in Liquid Medium

A bacterial suspension with a density that is equivalent to the 0.5 Mac Farland standard (10^8^ CFU/mL) was prepared from an 18 to 24 h bacterial culture. A stock solution was prepared by mixing (*v*/*v*) essential oil and DMSO (the chosen emulsifier). The dilution of the essential oil used is 70% (70% EO, 30% DMSO). A volume of 40 μL of the bacterial suspension was deposited in test tubes containing 4 mL of liquid culture medium. Then, we aseptically added the different volumes of the emulated essential oils, in order to obtain an increasing range of final concentrations of the essential oils from C1 to C9 that correspond respectively to 1, 4, 8, 16, 32, 64, 128, 256 and 512 μL/mL. A negative control tube containing only the suspension and the culture medium was prepared. Three repetitions were made for each test. The whole was vortexed for homogenization, and was then incubated in an oven at a temperature of 37 °C for 24 h. The MIC is the lowest essential oil concentration that can inhibit any growth that is visible to the naked eye after 24 h of incubation at 37 °C. In addition, the MBC was determined by inoculating 100 mL of each tube, which has a concentration that is greater than or equal to the MIC, into solid medium (MHA). The lowest essential oil concentration at which 99.99% of bacteria were eliminated after 24 h of incubation at 37 °C was the MBC. The calculation of the MBC/MIC ratio made it possible to determine the bactericidal (MBC/MIC < 4) or bacteriostatic (MBC/MIC ≥ 4) effect of the studied essential oils.

## 4. Statistical Analysis

The statistical analysis was performed by OriginPro 8.5 software. All data were expressed as means ± SD of a minimum of three replicates or measurements and werecompared by one-way ANOVA test, followed by the Tukey test. *P*-values less than or equal to 0.05 wereconsidered statistically significant.

## 5. Conclusions

The aim of this work was to characterize the chemical composition of the aerial part essential oil of *T. capitatus* and *T. broussonetii* Boiss from Morocco and to evaluate the antioxidant and antibacterial properties of the essential oils. From the results obtained, it seems that these two plants have virtues that can justify their important use in traditional medicine. The composition of the EOs partially explains the higher potential observed (antioxidant and antibacterial), especially the role of the carvacrol/thymol major compounds in the overall bioactivity. The antibacterial results require more attention as they showed excellent activities even against bacteria. From all of the above, we confirm the traditional use of those plants EO against infectious disease and we also suggest more advanced tests for more personalized applications either in the clinical as antibiotics or the industries fields as preservatives.

## Figures and Tables

**Table 1:** Chemical composition of *T. capitatus* and *T. broussonetii* EOs.

N°	Compounds	IK (Adams)	*Thymus* *capitatus*	*Thymus broussonetii*	Formula	M
1	α-Thujene	930	-	0.38	C_10_H_16_	136
2	α-pinene	939	0.54	0.54	C_10_H_16_	136
3	Camphene	954	0.14	-	C_10_H_16_	136
4	Octen-3-ol	979	0.49	0.71	C_8_H_16_ O	128
5	*β*-Pinene	979	-	0.09	C_10_H_16_	136
6	Myrcene	990	0.88	0.97	C_10_H_16_	136
7	α-Phellandrene	1002	-	0.15	C_10_H_16_	136
8	*p*-Mentha-1(7),8-diene	1004	0.25	-	C_10_H_16_	136
9	δ-3-Carene	1011	0.09	-	C_10_H_16_	136
10	α–Terpinene	1017	0.36	-	C_10_H_16_	136
11	*p*-Cymene	1026	10.58	6.21	C_10_H_14_	134
12	Limonene	1029	0.39	0.31	C_10_H_16_	136
13	*β*-Phellandrene	1029	-	0.19	C_10_H_16_	136
14	γ–Terpinene	1059	0.53	4.47	C_10_H_16_	136
15	(Z)-Sabinene hydrate	1070	-	0.33	C_10_H_18_	138
16	*m*-Cymene	1085	0.11	-	C_10_H_12_	132
17	Terpinolene	1088	-	1.2	C_10_H_16_	136
18	Linalool	1096	2.29	0.14	C_10_H_18_O	154
19	(E)-Sabinene hydrate	1098	-	0.16	C_10_H_18_O_2_	170
20	Terpinen-4-ol	1177	0.20	0.74	C_10_H_18_O	154
21	Borneol	1169	-	0.25	C_10_H_18_O	154
23	α-terpineol	1188	-	0.09	C_10_H_18_O	154
24	Carvacrol, methyl ether	1244	-	0.2	C_11_H_16_O	164
25	Thymol	1290	0.18	12.9	C_10_H_14_O	150
26	Carvacrol	1299	75	60.79	C_10_H_14_O	150
27	Terpinylisobutyrate	1473	0.14	-	C_14_H_24_O_2_	224
28	Piperitenone	1343	0.06	-	C_10_H_14_O	150
29	Eugenol	1359	0.15	-	C_10_H_12_O_2_	164
30	Trans-sobrerol	1374	0.1	-	C_10_H_18_O_2_	170
31	(*E*)-Caryophyllene	1419	1.61	4.15	C_15_H_24_	204
32	Aromadendrene	1441	-	0.90	C_15_H_24_	204
33	α-Humulene	1454	-	0.13	C_15_H_24_	204
34	9-epi-(e)-caryophyllene	1466	-	0.19	C_15_H_24_	204
35	Muurolene<γ->	1479	-	0.19	C_15_H_24_	204
36	Viridiflorene	1496	-	0.76	C_15_H_24_	204
37	*β*–Bisabolene	1505	0.79	0.47	C_15_H_24_	204
38	δ-Cadinene	1523	-	0.34	C_15_H_24_	204
39	(*E*)-iso-γ-Bisabolene	1524	0.28	-	C_15_H_24_	204
40	Thymohydroquinone	1555	0.69	-	C_10_H_14_O_2_	166
41	Epoxy caryophyllene	1583	1.50	0.16	C_15_H_24_O	220
42	Caryophylla-4(12),8(13)-dien-5α-ol	1640	0.10	-	C_15_H_24_O	220
43	Selina-3,11-dien-6-α-ol	1644	0.25	-	C_15_H_24_O	220
44	Germacra-4(15),5,10(14)-trien-1α-ol	1686	0.22	-	C_15_H_24_O	220
45	Geranyl benzoate	1959	0.56	-	C_17_H_22_O_2_	258
46	*cis*-Totarol, methyl ether	2237	-	0.23	C_21_H_32_O	300
Percentage of monoterpenes hydrocarbons					13.87	14.84
Percentage of oxygenated monoterpenes					79.3	75.98
Percentage of sesquiterpenes hydrocarbons					2.68	7.13
Percentage of oxygenated sesquiterpenes					2.63	0.39
Total identified (%)					98.48	98.34

**Table 2:** Antibacterial activities of *T. capitatus* and *T. broussonetii* EOs and of antibiotics (FOX30, TIM85, PRL100) using the disk diffusion method (mm).

Strains	*T. capitatus*	*T. broussonetii*	Antibiotics		
			FOX30	TIM85	PRL100
*Enterococcus faecalis*	23.6 ± 0.85 ^abc^	20.85 ± 0.75 ^abc^	0	0	9 ± 0.00
*S. aureus*	9.3 ± 0.35 ^abc^	8.55 ± 0.2 ^abc^	0	0	0
*Serratia fonticola*	49.8 ± 0.28 ^abc^	45.35 ± 0.65 ^abc^	0	0	0
*Acinetobacter baumannii*	22.6 ± 0.57 ^c^	20.7 ± 1.20 ^c^	21 ± 0.00	15 ± 0.00	10.5 ± 0.00
*Klebsiella oxytoca*	49.9 ± 0.14 ^abc^	48.05 ± 0.95 ^abc^	0	0	11 ± 0.00
*Klebsiella pneumoniae* sensitive	19.95 ± 0.07 ^bc^	14.15 ± 0.15 ^bc^	12 ± 0.00	0 ± 0.00	0
*E. coli* sensitive	45.2 ± 1.13 ^abc^	45.6 ± 0.4 ^abc^	22 ± 0.00	9 ± 0.00	0
*E. coli* resistant	15.5 ± 0.7 ^abc^	14.65 ± 0.35 ^abc^	0	0	0
*Enterobacter aerogenes*	28.7 ± 0.42 ^bc^	28.9 ± 0.3 ^bc^	20 ± 0.00	14 ± 0.00	10 ± 0.00

Data are expressed as mean ± SD. The experiment was performed in a minimum of three replicates. Significance level of at least *p* < 0.05 compared to FOX30 (^a^), TIM85 (^b^), PRL100 (^c^).

**Table 3:** Minimal inhibitory concentration (MIC), minimal bactericidal concentration (MBC) and the MBC/MIC report values (mg/mL) of *T. capitatus* and *T. broussonetii* EOs.

Strains	Essential Oils					
	*T. capitatus*			*T. broussonetii*		
	MIC	MBC	MBC/MIC	MIC	MBC	MBC/MIC
*E. aerogenes*	4 ± 0.000	80 ± 0.00	20 ± 0.003	8 ± 0.002	8 ± 0.003	2 ± 0.000
*S. aureus*	16 ± 0.005	32 ± 0.009	1 ± 0.001	32 ± 0.001	32 ± 0.00	1 ± 0.004
*E. faecalis*	4 ± 0.001	8 ± 0.001	2 ± 0.006	4 ± 0.001	4 ± 0.001	1 ± 0.001
*E. coli* sensitive	2 ± 0.005	4 ± 0.00	2 ± 0.00	2 ± 0.001	4 ± 0.002	2 ± 0.001
*E. coli* resistant	8 ± 0.002	32 ± 0.004	4 ± 0.001	16 ± 0.003	32 ± 0.007	1 ± 0.001
*K. oxytoca*	4 ± 0.003	8 ± 0.001	1 ± 0.002	4 ± 0.001	8 ± 0.002	1 ± 0.000
*S. fonticola*	4 ± 0.006	8 ± 0.003	1 ± 0.006	4 ± 0.007	8 ± 0.001	1 ± 0.000
*K. pneumoniae* sensitive	2 ± 0.00	4 ± 0.004	10 ± 0.001	2 ± 0.005	4 ± 0.008	1 ± 0.000
*A. baumannii*	4 ± 0.002	4 ± 0.00	20 ± 0.002	4 ± 0.008	8 ± 0.002	2 ± 0.003

Data are expressed as mean ± SD. The experiment was performed in a minimum of three replicates.

## Data Availability

Data are available upon request.
